# Fixation of Distal Radius Fractures Under Wide-Awake Local Anesthesia: A Systematic Review

**DOI:** 10.1177/15589447221109632

**Published:** 2022-07-26

**Authors:** Kyle Gouveia, Eric Harbour, Aaron Gazendam, Mohit Bhandari

**Affiliations:** 1 Division of Orthopaedic Surgery, McMaster University, Hamilton, ON, Canada; 2 School of Medicine, University of Limerick, Ireland

**Keywords:** WALANT, wrist, fracture/dislocation, diagnosis, distal radius, anesthesia, specialty, local anesthesia

## Abstract

**Background:**

The purpose of this systematic review was to analyze the available literature on fixation of distal radius fractures (DRFs) under wide-awake local anesthesia no-tourniquet (WALANT), and to examine postoperative pain scores and functional outcomes, operative data including operative time and blood loss, and the frequency of adverse events.

**Methods:**

Embase, MEDLINE, Web of Science, and SCOPUS were searched from inception until May 2022 for relevant studies. Studies were screened in duplicate, and data on pain scores, functional outcomes, and adverse events were recorded. Due to methodological and statistical heterogeneity, the results are presented in a descriptive fashion.

**Results:**

Ten studies were included comprising 456 patients with closed, unilateral DRFs, of whom 226 underwent fixation under WALANT. These patients had a mean age of 52.8 ± 8.3 years, were 48% female, and had a mean follow-up time of 11.6 months (range: 6-24). Operative time for WALANT patients averaged 60.4 ± 6.5 minutes, with mean postoperative pain scores of 1.4 ± 0.6 on a 10-point scale. Studies that compared WALANT to general anesthesia found shorter hospital stays with most WALANT patients being sent home the same day, decreased postoperative pain scores, and decreased costs to the healthcare system. No adverse events were reported for WALANT patients.

**Conclusions:**

A growing body of literature reports that for closed, unilateral DRF, surgical fixation under WALANT is a safe and effective option. It allows patients to have surgery sooner, with improved pain scores and good functional outcomes, with a very low incidence of adverse events.

## Introduction

Distal radius fractures (DRFs) represent one of the most common fracture types, affecting both the elderly and younger populations. Following hip fractures, DRFs are the most common fractures in the elderly, with an estimated incidence of 643 000 per year in the United States.^1^ Not surprisingly, this has resulted in significant financial burden, with estimates of Medicare expenditures ranging from $385 to $535 million USD annually.^2,3^ While many of these fractures can be treated nonoperatively, the proportion undergoing internal fixation has risen in the past 2 decades since the introduction of volar plating in the early 2000s.^4^

Surgical procedures for DRF are typically done under either a general anesthetic or regional anesthesia (RA) such as brachial plexus blockade along with the use of a tourniquet.^5^ However, many other hand and wrist surgical procedures are performed under strictly local anesthesia, allowing for decreased costs and improved patient experience.^6-8^ Additionally, proponents of wide-awake local anesthesia no-tourniquet (WALANT) technique cite the ability to assess the full active range of motion (ROM) intraoperatively as an advantage of the technique.^9^ The use of local anesthesia can potentially help to mitigate some of the risks of general anesthesia to an older and more frail patient population,^10^ which make up a significant proportion of DRFs. As well, in the setting of the ongoing COVID-19 pandemic, alternative anesthesia options are being given greater attention given that general anesthesia involves aerosol-generating procedures.^11^

While this anesthetic technique has typically been reserved for minor hand and wrist procedures such as carpal tunnel or trigger finger release,^12,13^ it has recently been employed for larger surgical procedures such as cubital tunnel release,^14^ as well as fixation of fractures of the ankle, clavicle, and olecranon.^15-17^ Furthermore, recent literature has described the use of local anesthesia for operative fixation of DRFs.^18^ Also notable is that procedures done under local anesthesia can often be done in a procedure room and do not require dedicated operating room (OR) time,^19,20^ which is especially important given the current pandemic has created a backlog of surgeries and has made OR time an even more valuable resource.^21^

The purpose of this systematic review was to analyze the available literature on the fixation of DRFs under WALANT, and to examine surgical and functional outcomes, as well as the frequency of complications or adverse events. Furthermore, where applicable we will compare DRF fixation under local anesthesia to other forms of anesthesia used in the surgical fixation of these fractures.

## Methods

This systematic review was conducted in accordance with the Preferred Reporting Items for Systematic Reviews and Meta-Analyses (PRISMA) guidelines for conducting and reporting systematic reviews.^22^ The study protocol was registered prospectively on The International Prospective Register of Systematic Reviews (PROSPERO # CRD42020214111).

### Literature Search

The online databases MEDLINE, Embase, Web of Science, and SCOPUS were searched from database inception to May 18, 2022, for literature pertaining to the surgical fixation of DRFs under local anesthesia. Search terms included DRFs and their various eponyms, as well as various types of local anesthesia. A full search strategy is available in Table A1.

### Study Screening

Studies identified were screened at the title and abstract, as well as full-text stage by 2 blinded reviewers (KG and EH), using the online software Rayyan QCRI (2010, Qatar Computing Research Institute, Doha, Qatar). Any discrepancies at the title and abstract stage were resolved with automatic inclusion in the next stage of screening. At the full-text stage of screening any discrepancies were discussed and resolved by agreement between the reviewers. The references of included studies subsequently underwent manual screening to identify any additional articles which may have been excluded in the initial search strategy.

### Assessment of Study Eligibility

The inclusion and exclusion criteria were defined *a priori*. Inclusion criteria included: (1) investigated the operative fixation of DRFs; (2) fixation was done under local anesthesia without a tourniquet; (3) studies were of Level I-IV evidence; and (4) studies were published in the English language. Studies were excluded if: (1) they involved other forms of anesthesia including general anesthesia (GA), intravenous RA (Bier block), or nerve blocks; (2) they involved local anesthesia only as an adjunct to GA; (3) they were nonhuman studies; and (4) if they were technique papers, editorial commentaries, review articles, or other papers without extractable primary data.

### Data Extraction

Two reviewers (KG & EH) extracted data from included studies independently and in duplicate into a spreadsheet designed *a priori* and piloted prior to use. Extracted data included study characteristics, patient demographics, local anesthetic technique, pain and functional outcomes, and the frequency of adverse events or complications. Data on comparator groups such as GA or brachial plexus block were also extracted if available.

### Study Appraisal

The risk of bias in the included randomized controlled trials (RCTs) was assessed using the Revised Cochrane Risk of Bias Tool (Cochrane Risk of Bias 2.0).^23^ The methodological quality of nonrandomized studies was evaluated using the Methodological Index for Non-Randomized Studies (MINORS) criteria.^24^ Using the items on the MINORS checklist, noncomparative studies can achieve a maximum score of 16, while comparative studies can achieve a maximum score of 24.

### Statistical Analysis

Due to high statistical and methodological heterogeneity, a meta-analysis could not be performed, and the results are summarized descriptively. Patient demographics and descriptive statistics including means, standard deviations, and ranges are presented where applicable.

## Results

### Literature Search

**Figure 1.**
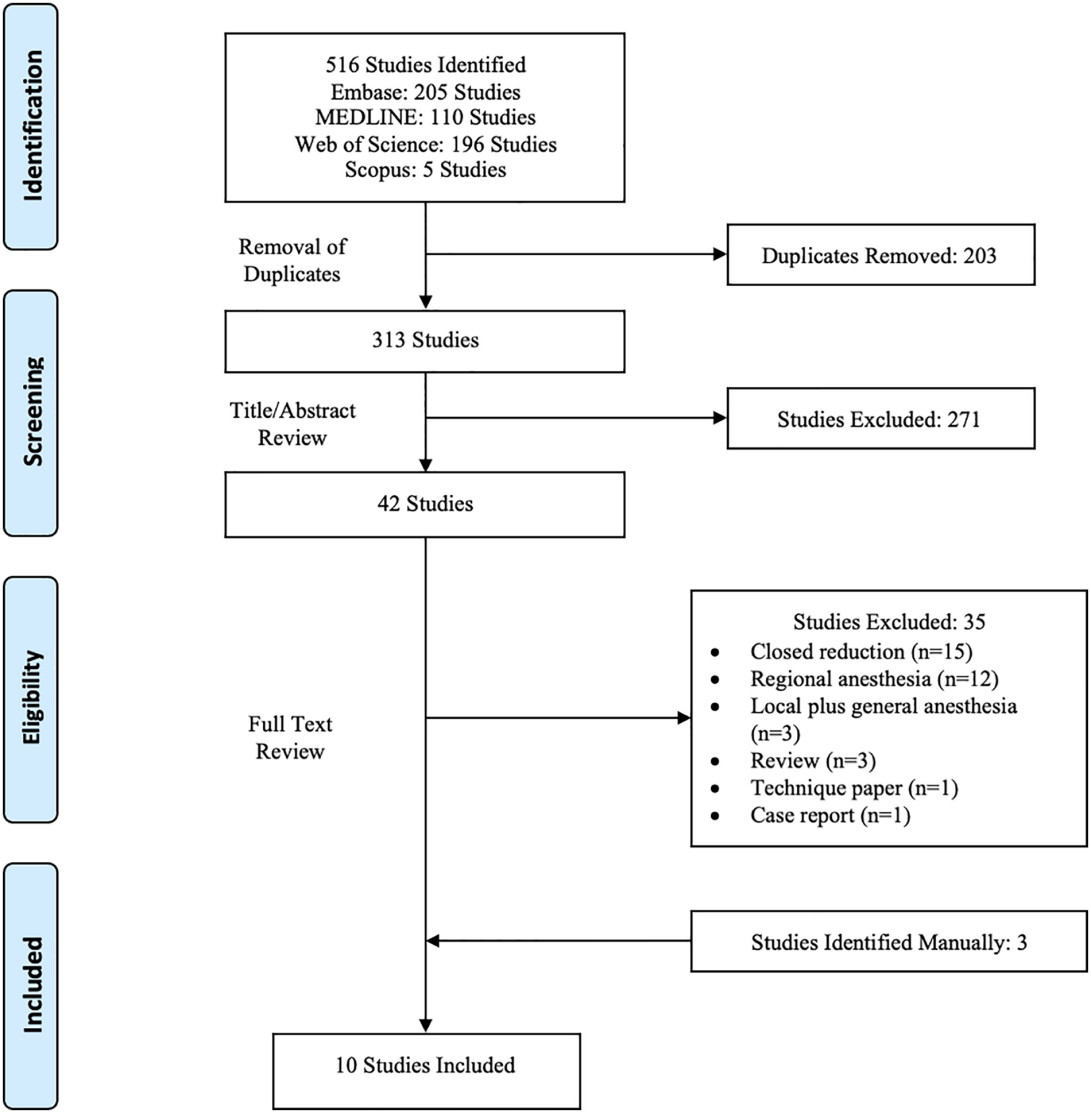
PRISMA flow diagram outlining the search and screening process.

The initial literature search yielded 516 studies, which after removal of duplicates was reduced to 313. Systematic screening and assessment of eligibility resulted in 10 full-text studies that satisfied inclusion criteria. A PRISMA flow diagram detailing the search and screening process is displayed in Figure 1.

### Study Quality

All 10 of the included studies were published from 2018 to 2022 (Table 1). Of the 10 included studies, there were 4 case series (Level IV evidence),^25-28^ 2 retrospective cohorts (Level III evidence),^29,30^ 1 case-control study (Level III evidence),^31^ 2 prospective cohorts (Level II evidence),^32,33^ and 1 RCT (Level I evidence).^34^ The mean MINORS score for the 4 noncomparative studies was 7, and for the 3 comparative studies it was 17. The lone RCT was found to raise some concerns according to the Revised Cochrane Risk of Bias Tool in the domains of missing outcome data and selection of reported result.

**Table 1.** Characteristics of Included Studies.

Study	Journal	Study design	Level of evidence	Number of LA patients	M/F	Mean age	Mean follow-up (MO)
Abitbol et al^33^	*Hand Surgery and Rehabilitation*	Prospective cohort	II	21	6/15	66.7	6
Amir et al^25^	*Malaysian Orthopedic Journal*	Case series	IV	5	NR	NR	NR
Dukan et al^32^	*Journal of Hand Surgery* [European Volume]	Prospective cohort	II	15	11/4	53 4.2	6
Huang et al^26^	*Journal of Orthopedic Surgery and Research*	Retrospective cohort	III	24	9/15	60.9 (20-88)	15.1 (12-24)
Huang et al^29^	*Orthopedics*	Retrospective cohort	III	21	8/13	65.29 ± 15.47	12
Liu et al^31^	*Journal of Clinical Medicine*	Case-control study	III	20	3/17	62.2 (13.5)	NR
Orbach et al^27^	*Journal of International Medical Research*	Case series	IV	5	5/0	40.2 ± 17.2	NR
Tahir et al^28^	*Journal of Pakistan Medical Association*	Case series	IV	40	22/18	45.23 ± 12.22	10.65 ± 3.54
Tahir et al^34^	*Bone and Joint Research*	Randomized controlled trial	I	55	31/24	46.6 ± 10.81	12
Yi et al^30^	*Journal of Hand Surgery Global Online*	Retrospective cohort	III	20	19/1	41 (18-73)	NR

*Note.* NR = not reported; MO = months; LA = local anesthesia.

### Patient Characteristics

There were a total of 456 patients analyzed in the 10 included studies. Of these, 226 underwent fixation of a DRF under local anesthesia, and more specifically all were done using a WALANT approach (Table 1). Of these 226 patients, 48% were female (107/221), and the mean age was 52.8 ± 8.3 years. Postoperative follow-up ranged from 6 to 24 months, with a mean follow-up time of 11.6 months.

All patients had a closed, unilateral DRF. All studies that reported patient exclusion criteria indicated that patients with open injuries, multiple fractures, or an allergy to the local anesthetic were not candidates for DRF fixation under WALANT. According to the AO/Orthopaedic Trauma Association (AO/OTA) classification, there were a total of 29 type A2 fractures, 28 type A3 fractures, 12 type B1 fractures, 10 type B2 fractures, 20 type B3 fractures, 21 type C1 fractures, 30 type C2 fractures, and 10 type C3 fractures. One additional study simply reported that all fractures were dorsally displaced and 12 of 15 were intraarticular,^32^ while another described its 5 fractures as 2 volar Barton fractures, 1 Smith fracture, 1 dorsal die-punch fracture, and 1 comminuted intraarticular fracture.^27^ The majority of patients underwent open reduction and internal fixation (ORIF) with a volar locking plate via a modified Henry or flexor carpi radialis (FCR) based approach. This accounted for 98% (221/226) of patients, while the other 5 patients underwent ORIF with a plate via a dorsal-based approach.

### Anesthetic Technique

As previously mentioned, all included studies utilized the WALANT technique. Anesthetic solutions primarily consisted of 1% or 2% lidocaine, and all used lidocaine with epinephrine. Three studies (41 WALANT patients)^27,32,33^ reported infiltrating local anesthesia in a preoperative holding area prior to being transferred to the operating room. Full details of the anesthetic technique were reported by all 8 studies and are available in Table 2. Four of 10 studies began with an initial hematoma block of 3-5 cc,^26,28,29,34^ and all reported use of copious local anesthesia in the skin and subcutaneous tissue around the incision. Lastly, all studies described some form of deep local anesthetic infiltration, whether beneath the pronator quadratus (5 studies),^26,28,29,31,34^ or from various skin-marked injection points with the needle on bone (5 studies).^25,27,30,32,33^ Of the 7 studies that reported their postoperative protocol, 4 studies^27,28,32,34^ allowed immediate full ROM as tolerated and the remaining 3^26,29,33^ utilized a 1- to 2-week period of immobilization.

**Table 2.** Fracture Types and Fixation Techniques, as Well as Local Anesthesia Techniques of Included Studies.

Study	Fracture characteristics	Fixation details	LA solution	Anesthetic technique
Abitbol et al^33^	Dominant side 11/21 Intra-articular 5/21	Volar plating.	40 ml lidocaine 1% with epinephrine buffered with 4 ml sodium bicarbonate 8.4%	Incision infiltrated with 10 ml, 5 ml under skin, and 5 ml deep to fascia. Then, 20 ml divided between 2 points on bone at the radial edge of the radius. Lastly, 10 ml injected directly into fracture site.
Amir et al^25^	NR	Volar plating.	Solution of 50 ml normal saline, 50 ml 2% lidocaine, 10 ml 8.4% sodium bicarbonate, and 1 ml adrenaline 0.18%	Injection along skin layer and subperiosteally.
Dukan et al^32^	100% (15/15) of fractures were dorsally displaced, 80% (12/15) were intraarticular	Volar plating.	Preinjection skin anesthetic cream (Emla cream 5%) followed by a solution of 50 ml normal saline, 50 ml 1% lidocaine with epinephrine, and 8 ml 8.4% sodium bicarbonate	There were 15 injection points marked, 5 anterior, 5 radial, and 5 posterior. A total of 5 ml was injected. subcutaneously along the incision site and at each marked point 2 ml was injected subcutaneously and 4 ml with the needle on bone.
Huang et al^26^	6 AO/OTA type A2, 4 type A3, 3 type B2, 5 type B3, 2 type C1, 3 type C2, and 1 type C3	Volar plating in 21 patients, dorsal plating in 3 patients.	40 ml of 1% lidocaine with 1:40 000 epinephrine	Initial hematoma block with 3-5 ml, followed by 5-10 ml subcutaneously at the operative site. An additional 5 ml was injected beneath the PQ (or extensor retinaculum for the dorsal approach).
Huang et al^29^	3 AO/OTA type A2, 6 type A3, 5 type B2, 3 type C1, and 4 type C2	Volar plating.	40 ml of 1% lidocaine with 1:40 000 epinephrine	Initial hematoma block with 3-5 ml, followed by 5-10 ml subcutaneously at the operative site. An additional 5 ml was injected beneath the PQ.
Liu et al^31^	14 AO/OTA Type A, 3 AO/OTA Type B, 3 AO/OTA Type C	Volar plating.	Approximately 40 ml of 1% lidocaine with 1:100 000 epinephrine.	Incision area infiltrated with ~15 ml to start. Next, 10 ml was injected through the PQ intended to anesthetize the volar periosteum, followed by 10 ml for the dorsal periosteum, getting patients to pronate the forearm. Lastly, 2-3 ml was used over the radial styloid to prepare for wire placement.
Orbach et al^27^	2 volar Barton, 1 Smith, 1 dorsal “die-punch”, and 1 comminuted intra-articular	Volar plating in 3 patients, dorsal plating in 2 patients.	Solution of 50 ml normal saline and 50 ml of 1% lidocaine with 1:1 000 000 epinephrine	A total of 8 points were marked depending on volar or dorsal approach. A total of 15 ml was injected subcutaneously along the incision site and at each marked point 2 ml was injected subcutaneously and 4 ml with the needle on bone.
Tahir et al^28^	13 AO/OTA type A2, 11 type A3, 5 type B3, 6 type C1, 4 type C2, and 1 type C3	Volar plating	Solution of 50 ml normal saline and 50 ml of 1% lidocaine with 1:100 000 epinephrine	Initial hematoma block with 3-5 ml, followed by 5-10 ml subcutaneously at the operative site. An additional 5 ml was injected beneath the PQ.
Tahir et al^34^	6 AO/OTA type A2, 5 type A3, 11 type B1, 5 type B3, 7 type C1, 15 type C2, and 6 type C3	Volar plating	Solution of 50 ml normal saline and 50 ml of 2% lidocaine with 1:1 000 000 epinephrine	Initial hematoma block with 3-5 ml, followed by 5-10 ml subcutaneously at the operative site. An additional 5 ml was injected beneath the PQ.
Yi et al^30^	1 AO/OTA type A2, 2 type A3, 1 type B1, 2 type B2, 5 type B3, 3 type C1, 4 type C2, and 2 type C3	Volar plating	Solution of 50 ml normal saline, 50 ml 2% lidocaine, 10 ml 8.4% sodium bicarbonate, and 1 ml adrenaline 0.18%	A total of 3 points were marked over the radial border of the radius. First, 10 ml was injected along the incision site. Following this, at each marked point 10 ml was used subperiosteally (2 ml radial, and 4 ml volar and dorsal).

*Note.* OTA = Orthopaedic Trauma Association; LA = local anesthesia; NR = not reported; PQ = pronator quadratus.

### Operative Data

Operative data including anesthetic time, operative time, and estimated blood loss are available in Table 3. The mean total anesthetic time for the WALANT patients from arrival in the operating room to procedure start was reported in 2 studies (76 patients),^29,34^ and was found to be 23.7 ± 4.0 minutes. Operative time for WALANT patients was reported by 8 studies (216 patients),^26,28-34^ and averaged 60.4 ± 6.5 minutes. Lastly, the mean estimated blood loss in those patients undergoing WALANT was reported by 6 studies (180 patients),^26,28-31,34^ and was found to be 22.3 ± 6.4 ml.

**Table 3.** Major Outcomes of Included Studies.

Study	Group	Number of patients	Anesthetic time (min)	Operative time (min)	Blood loss (ml)	VAS pain POD1	Flexion ROM	Extension ROM	Adverse events
Abitbol et al^33^	WALANT	21	NR	36	NR	NR	75	70	0
	RA	20	NR	37	NR	NR	74	68	0
Amir et al^25^	WALANT	5	NR	NR	NR	NR	NR	NR	0
Dukan et al^32^	WALANT	15	NR	38	NR	0.8 (1.2)*	NR	NR	0
	LRA	30	NR	31	NR	1.1 (0.9)*	NR	NR	0
Huang et al^26^	WALANT	24	NR	64.3 (45-85)	18.9 (5-30)	1.6 (1-3)	69.6 (55-80)	57.4 (45-70)	0
Huang et al^29^	WALANT	21	25.38 (4.59)	68.10 (9.28)	22.62 (6.82)	1.95 (0.67)	67.14 (9.95)	50.24 (9.28)	0
	GA	26	37.31 (11.16)	64.42 (10.42)	8.62 (9.23)	3.27 (1.28)	71.35 (8.19)	49.42 (6.22)	NR
Liu et al^31^	WALANT	20	NR	57.8 (16.4)	14.2 (13.1)	NR	NR	NR	0
	GA	20	NR	63.8 (20.6)	6.6 (5.9)	NR	NR	NR	0
Orbach et al^27^	WALANT	5	NR	NR	NR	NR	NR	NR	0
Tahir et al^28^	WALANT	40	NR	62.5 (9.26)	13.5 (6.81)	1.47 (0.81)	64.0 (5.08)	53.12 (5.39)	0
Tahir et al^34^	WALANT	55	23.0 (3.85)	61.3 (9.28)	23.4 (8.50)	1.2 (0.62)	65.9 (6.01)	54.8 (6.45)	0
	GA	56	33.7 (5.81)	68.8 (14.97)	11.5 (4.25)	3.0 (1.24)	64.3 (4.47)	52.9 (4.45)	3
	Bier block	58	30.2 (4.67)	65.5 (12.61)	14.0 (4.89)	2.2 (1.35)	64.4 (4.92)	53.4 (4.95)	3
Yi et al^30^	WALANT	20	NR	86	49	NR	NR	NR	0
	GA	20	NR	102	63	NR	NR	NR	3

*Note.* VAS = visual analogue scale; WALANT = wide-awake local anesthesia no-tourniquet; NR = not reported; POD1 = postoperative day 1; ROM = range of motion; LRA = loco-regional anesthesia; GA = general anesthesia; RA = regional anesthesia. *VAS pain at 6 weeks.

### Hospital Stay and Patient Satisfaction

The majority of patients went home the same day after DRF fixation under WALANT. Of the 6 studies that reported this, 4 had all WALANT patients go home the same day,^27,28,32,34^ while 1 had half of all patients (12/24) receiving surgery as outpatients,^26^ and 1 kept all patients overnight to monitor for adverse events of local anesthesia.^30^ In the 3 studies that reported mean hospital stay,^29,30,34^ the mean for WALANT patients was 0.6 ± 0.4 days, while it was 1.7 ± 0.6 days for GA. Two studies (210 patients)^33,34^ reported on patient satisfaction, and both found that patient satisfaction was significantly higher in the WALANT group compared to alternative forms of anesthesia. Furthermore, Tahir et al^34^ found that 53 of 55 (96.4%) of the WALANT patients indicated they would undergo the same procedure again.

### Pain and Functional Outcomes

Major postoperative outcomes are listed in Table 3. Overall, postoperative pain was mild, with an average visual analogue scale (VAS) pain score of 1.4 ± 0.6 on postoperative day 1 (POD1). The most commonly reported functional outcome scores were the Quick Disabilities of the Arm, Shoulder, and Hand (QuickDASH) score (5 studies, 155 WALANT patients),^26,28,32^-^34^ as well as the Mayo Wrist Score (3 studies, 116 WALANT patients).^28,29,34^ At final follow-up, which ranged from 6 to 24 months, these averaged 9.5 ± 3.1 and 84.8 ± 6.1, respectively. Postoperative ROM at final follow-up was reported by 5 studies (161 WALANT patients),^26,28,29,33,34^ with wrist flexion averaging 67.3º ± 4.6º and extension 56.2º ± 4.8º.

### Adverse Events and Time to Radiographic Union

Overall, of the 226 patients who were to undergo DRF fixation under local anesthesia, conversion to a GA was reported in 2 studies (3 patients, 1.3% overall), and was due to patient anxiety in all cases.^26,34^ Last, no adverse events or complications were reported for any patient in the WALANT group, while in the groups with other anesthesia options (GA, Bier block), there were a total of 9 adverse events. Most commonly these were postoperative nausea and vomiting (n = 3), as well as tourniquet palsy (n = 2), and mild wound inflammation (n = 2). Time to union was reported by 3 studies (116 WALANT patients),^28,29,34^ and averaged 16.3 ± 2.7 weeks. One additional study simply reported that all fractures were united by the 6-month follow-up visit.^32^

### Comparative Studies

Four studies (238 patients)^29-31,34^ compared WALANT to GA, with 1 also having a group receive a Bier block, and 2 additional studies (86 patients)^32,33^ compared it to RA in the form of an axillary brachial plexus block. Among these comparative studies, no significant differences in patient demographics or fracture patterns were noted between the WALANT and comparator groups. In their study that compared WALANT to a brachial plexus block, Dukan et al^32^ found that there was no significant difference in postoperative pain scores; however, early ROM was greater in the WALANT group. These differences were negligible at the 6-month follow-up. In these patients, early QuickDASH scores also favored WALANT, and WALANT patients returned to work significantly earlier. Abitbol et al^33^ also compared RA to WALANT, and also found earlier return to work in the WALANT group as well as earlier recovery of wrist function via the QuickDASH score.

Of the 4 studies comparing WALANT to GA, 2 found lower postoperative pain scores in the WALANT group,^29,34^ and both studies that reported hospitalization time postoperatively found that WALANT patients returned home faster.^29,30^ Furthermore, both studies reporting on wait times to surgery found that WALANT patients got their surgeries significantly sooner than patients requiring a GA.^30,34^ In a slightly different approach, Liu et al^31^ investigated hemodynamics and found less fluctuation in mean arterial pressure (MAP) with WALANT compared to GA. Last, Tahir et al^34^ found that there was a significant difference between WALANT and GA with regard to overall costs to the healthcare system, with fixation under GA costing $630.63 (SD ± $114.77) and WALANT fixation costing $428.50 (SD ± $77.71), despite the need for conversion to GA in 2 WALANT patients.

## Discussion

The key finding of this review is that for the closed, isolated, unilateral DRF, surgical fixation under local anesthesia is a safe and effective option. Operative time for WALANT patients averaged 60.4 ± 6.5 minutes, with mean blood loss of 22.3 ± 6.4 ml and mean postoperative pain scores of 1.4 ± 0.6 on a 10-point scale. Furthermore, studies that compared WALANT to GA found shorter hospital stays, decreased postoperative pain scores, and decreased costs to the healthcare system. Last, no adverse events were reported for patients undergoing WALANT for operative DRF fixation.

Findings of this review appear to support WALANT as an alternative anesthetic technique for DRF fixation. While having comparable operative times and blood loss to traditional techniques,^29,35,36^ WALANT also resulted in low postoperative pain scores as well as positive functional outcomes. Tourniquet-related pain is a known contributor to postoperative pain following upper extremity surgery.^37^ The avoidance of a tourniquet when utilizing the WALANT may contribute to the low postoperative pain scores.^26,28,29,32,34^ Regardless of positive results in terms of pain and functional outcome scores, the greatest benefit of WALANT for DRFs may relate to decreased costs and shorter waiting times to surgery. With the increasing proportion of DRFs treated operatively, more of these patients are spending lost time waiting for surgery.^38^ WALANT for DRFs create the opportunity to perform fracture fixation without the services of an anesthetist, which can be the limiting factor for the on-call surgeon. Tahir et al^34^ found that patients waiting for surgery under GA waited nearly a week, compared to an average of 1.22 days for WALANT patients. This, combined with earlier return to work postoperatively, lead to WALANT patients missing an average of 7.8 working days total, compared to 20.1 for patients operated on under GA.^34^

Despite the advantages of WALANT for DRF fixation, it is not universally applicable to fracture patients. As with many studies in orthopedics, there were strict inclusion and exclusion criteria for patients such that only those with closed, unilateral, and isolated DRFs were included. This is quite understandable however, as patients with concomitant injuries or requiring irrigation and debridement for open fractures would not be amenable to WALANT. However, open DRFs are quite rare,^39^ and the population examined in studies included in this review represents a majority of DRFs. Additionally, this supports the notion that the benefits of WALANT for DRFs is likely for these patients with isolated injuries and with less cost to the system.^34^ Another potential drawback is the possibility of conversion to GA. While only occurring in 2 studies and with an overall incidence of 1.3%, the potential for conversion to GA indicates that an anesthesiologist may need to be aware of the case. However, as with many WALANT procedures, proper patient selection is essential and can significantly decrease the chances of conversion. The primary concern referenced with regard to selection is patient anxiety, though emerging strategies such as the use of noise-canceling headphones are being investigated.^40^ Lastly, patient movement can make procedures technically difficult at times, though the ability to test ROM intraoperatively is invaluable.

While DRF fixation under WALANT has both its advantages as well as drawbacks, the potential setting of this procedure plays a role in determining its utility and cost-saving effectiveness. While many hand surgery procedures under local anesthesia can be done in an emergency department or procedure room setting, fixation in an operating room setting was reported universally in this review. Literature has shown that hand and wrist procedures such as carpal tunnel release done in a procedure room rather than operating room results in no increased risk for infection while decreasing costs dramatically, though this lack of difference in infection rates has not been demonstrated for fracture fixation under WALANT.^41^ Furthermore, hand fracture fixation in a procedure room setting in the form of closed reduction and percutaneous pinning has also been demonstrated, with it being roughly one-quarter to one-third of the cost of the procedure in an operating room.^19,20^ However, a survey found that the primary barrier to hand fracture fixation in a procedure room setting is the absence of necessary equipment,^20^ and this is likely the same case for wrist fracture fixation. Open reduction and internal fixation of DRFs requires specialized equipment including drills, plates, screws, and fluoroscopy, precluding the ability to perform these procedures under WALANT in a procedure room setting. Furthermore, the need to potentially convert to GA makes attempting this operation in a procedure room quite precarious. Nevertheless, the potential to perform these procedures without an anesthesiologist has shown to decrease costs and reduce the wait time for surgery.^30,34^ In the setting of the ongoing pandemic, with surgical backlogs and limited OR time, the ability to perform these procedures without an operating room would be highly advantageous to provide efficient fracture care.

In this review high quality evidence was limited, with only 6 of 10 studies being comparative, and only 1 RCT. While recent research has begun to compare RA in the form of brachial plexus blockade to GA for DRFs,^5,42^ local anesthesia is a relatively novel technique and the literature is in its infancy. However, results of comparative studies in this review were generally positive. There was no increased risk of complications or adverse events with WALANT, and actually there were less anesthetic related adverse events when compared to GA.^30,34^ Moreover, in addition to decreased costs, WALANT lead to better early postoperative ROM and earlier return to work than GA in comparative studies.^29,32,34^ Early ROM is key in upper extremity injuries to prevent stiffness,^43^ and WALANT appears to support this goal following DRFs. Lastly, in comparative studies that reported adverse events, there were no adverse events in WALANT patients.^29,30,32,34^ This is in contrast to reported adverse events in the GA groups including postoperative nausea and vomiting. It should be noted that digital necrosis following the use of local anesthesia with epinephrine has been reported in the literature; however, this decrease in perfusion is almost always reversible.^44^ No adverse events related to local anesthesia use were reported in the included studies, which may be attributable to the use of local anesthesia in the more forgiving environment of the wrist rather than fingers.

The strengths of this systematic review include its rigorous methodology and comprehensive literature search. It is an extensive overview of the available literature on an alternative form of anesthesia for fixation of DRF. Moreover, while limited comparative evidence was available, this review offered insight into comparing local anesthesia to the more traditionally used GA for fixation of these fractures. This review allows clinicians to consider WALANT anesthesia in DRFs as a potential to decrease costs and surgical wait times, and improve patient outcomes.

The primary limitations of this review stem from the quantity and quality of evidence available on the topic, with a total of only ten studies included and only 1 study representing Level I evidence, which had methodological concerns of its own. While there were other larger comparative studies, 4 of the included studies were case series. These nonrandomized studies were likely subjected to a degree of patient selection bias, with patients more suitable for fracture fixation under local anesthesia selected for that anesthetic option. Patients who were anxious about WALANT were simply excluded from most studies along with patients excluded for other reasons, and so we cannot make conclusions about what proportion of patients were seen as unsuitable for WALANT. In addition to patient selection bias, these retrospective and nonrandomized studies had a great deal of variability when it came to outcome reporting, and were limited in their follow-up. While only 10 studies were included, all 10 were from 2018 or later, showing evidence that this topic is an emerging one. Lastly as a limitation, the relative lack of comparative studies precluded our ability to conduct a meta-analysis, though we did choose to report on comparative studies separately.

## Appendix

**Table A1.** Sample MEDLINE search strategy.

1. Exp Radius Fractures/su [Surgery]
2. Distal radius fracture*
3. Distal radial fracture*
4. Colles fracture*
5. Distal radius
6. Radius fracture*
7. Smith fracture*
8. Barton fracture*
9. Chauffeur* fracture*
10. 1 OR 2 OR 3 OR 4 OR 5 OR 6 OR 7 OR 8 OR 9
11. Exp Anesthesia, Local/
12. Exp Anesthetics, Local/
13. Local anesthe*
14. Lidocaine OR Marcaine OR Bupivacaine OR Levobupivacaine OR Lignocaine OR Prilocaine OR Ropivacaine OR Mepivacaine
15. WALANT or wide-awake local anesthesia no tourniquet
16. 11 OR 12 OR 13 OR 14 OR 15
17. 10 AND 16
18. Limit 17 to: Humans
